# The Composition and Organization of Cytoplasm in Prebiotic Cells

**DOI:** 10.3390/ijms12031650

**Published:** 2011-03-03

**Authors:** Jack T. Trevors

**Affiliations:** School of Environmental Sciences, University of Guelph, Ontario, N1G 2W1, Canada; E-Mail: jtrevors@uoguelph.ca; Tel.: +1-519-824-4120; Fax: +1-519-837-0442

**Keywords:** bacteria, prebiotic cytoplasm, genetic instructions, hydrogel, origin of cytoplasm, spatial organization

## Abstract

This article discusses the hypothesized composition and organization of cytoplasm in prebiotic cells from a theoretical perspective and also based upon what is currently known about bacterial cytoplasm. It is unknown if the first prebiotic, microscopic scale, cytoplasm was initially contained within a primitive, continuous, semipermeable membrane, or was an uncontained gel substance, that later became enclosed by a continuous membrane. Another possibility is that the first cytoplasm in prebiotic cells and a primitive membrane organized at the same time, permitting a rapid transition to the first cell(s) capable of growth and division, thus assisting with the emergence of life on Earth less than a billion years after the formation of the Earth. It is hypothesized that the organization and composition of cytoplasm progressed initially from an unstructured, microscopic hydrogel to a more complex cytoplasm, that may have been in the volume magnitude of about 0.1–0.2 μm^3^ (possibly less if a nanocell) prior to the first cell division.

## 1. Introduction

One of the enigmas in origin of life research is to understand the origin and composition of the first bacterial cytoplasm, genetic instructions, and then the emergence of spatially organized [1], energy transducing [2], living bacterial cells capable of growth/division and subsequent evolution [3–12]. Bacteria and bacterial cytoplasm, once thought of, and described as sacs of nucleic acids, ribosomes, lipids, proteins, amino acids and ions, are now better understood as spatially organized and regulated molecular biosystems, with DNA codes of instructions [13,14]. It is now known that bacteria have spatial organization [14] including DNA compaction, RNA, cytoskeleton structures, numerous specific cell division proteins and thousands of ribosomes (e.g., 10,000–12,000 per cell and up to 72,000 during rapid exponential growth) present in volumes ranging from about 0.2 (or even less) to several μm^3^, depending on the species and environmental growth conditions. The cell volumes depend upon the different species of bacterial cells and their stage of growth, under different and often rapidly changing environmental conditions. The cell cytoplasm can be, for example 60–70% of the total cell volume, and cannot be understood as a simple aqueous solution, but better as a structured gel environment [15,10]. The bacterial cell environment can be considered organized into three categories or zones [14]. These are (1) the nucleoid zone consisting of nucleic acid/protein complexes, (2) the structural zone consisting of cytoskeleton proteins and (3) the metabolic zone consisting of the space between categories one and two [14].

One of the open questions for scientists to ask and elucidate is the composition and organization of prebiotic cytoplasm, as experimentation and observations are difficult, and some are even impossible to conduct at this time. This may change with more pioneering research on the possibility of synthetic microbial life capable of cell division.

Coupled with this question on the origin of the precytoplasm is the origin of a stable boundary structure; a semipermeable, continuous, prebiotic membrane capable of enclosing precytoplasm in the magnitude of about a μm^3^ or even less, for prebiotic and possibly living nanocells. Other features in the organization and composition of prebiotic cytoplasm would be spatial molecular crowding in the small precytoplasmic volume, and the short diffusion distances encountered in the absence of any active transport systems. Diffusion has its limitations, as bacterial cell sizes would need to be smaller than present-day bacteria if active transport processes were not present. The bacterial cell size has to be small enough to allow some diffusion processes, but large enough to contain the cytoplasm and all necessary molecules, macromolecules and ions at non-limiting concentrations. The advantages of a crowded microscopic cytoplasm would be that the first prebiotic cytoplasm and the subsequent living cell(s) would only require less than 1 μm^3^ of cytoplasm, and the diffusion distances would be minimal. Spatial molecular crowding would be an advantage in the organization of the first, stable, pre-biotic cells.

Table 1 summarizes some possible features of the composition and organization of primitive cytoplasm and cytoplasm in the first living cell(s) capable of growth and division. It is also plausible to hypothesize the origin or source of the first primitive cytoplasm in prebiotic cells and that the cytoplasm in the first cell(s) capable of growth and division progressed from a simple unstructured precytoplasm to a more structured or organized cytoplasm. This would be a plausible progression when organizing cytoplasm from geochemicals (e.g., composed of carbon, nitrogen, iron, possibly polycyclic aromatic hydrocarbons and whatever elements and compounds were present on the early Earth; and some likely unknown if they were present) water and even possibly oily hydrocarbons or amphiphilic compounds (e.g., decanoic acid is a present day example). The exact composition of the precytoplasm is of course not known. This is an immense knowledge gap where plausible hypotheses and additional research are still required. The gel like structure(s) may have been entirely water-based, hydrocarbon-based hydrophobic, or amphiphilic compounds, or a combination of the two different chemical phases (e.g., hydrophobic and hydrophilic) interacting (or mixing). The presence of the first molecules and organizing polymers would influence and even control the water dynamics during precytoplasm organization. One immense information gap is that the exact composition of the precytoplasm is not known and can only be hypothesized at this time.

## 2. Will the Origin and Composition of the First, Unstructured Cytoplasm Ever Been Known?

The origin of bacterial life is an immense enigma from an experimental perspective as bacterial fossils and molecular signatures (e.g., DNA, RNA, lipids, proteins) are not always readily detectable for research. Ancient DNA can be recovered from some environmental samples; however, this has not lead to new knowledge on the origin of the first genetic material. In addition, prebiotic cytoplasm had to originate from inanimate geochemicals and water via mechanisms [5] that are currently not understood and generally agreed upon.

The origin of bacterial life is generally placed in water, as all life requires water. This is not entirely correct as bacteria can be freeze-dried and survive for many years. However, life does require water to metabolize and reproduce, based on our current knowledge. The molecular machinery required for life did not evolve without water as the solvent. Today, we know that microorganisms can be recovered from crude oil samples, but it is not known if they only survive in this crude oil substrate, or if growth and cell division are possible, especially at the interfaces between oil and water and/or in emulsions.

The origin of life on the Earth likely occurred under anaerobic, but extreme conditions by comparison to the present-day conditions. In an earlier article, it was hypothesized that a prebiotic gel that made the transition to a bacterial biofilm, and a bacterial mat, as a plausible progression and environment for the origin of life [20]. The events in the origin of the first bacterial cell(s) were hypothesized to be a prebiotic gel on or between a mineral surface (e.g., montmorillonite can be hypothesized) making the transition to a living biofilm or biosystem of bacterial cells, capable of growth and division, and subsequent Darwinian evolution via natural selection, gene transfer and mutational events.

In this article, an attempt to understand the composition and structure of primitive cytoplasm has been undertaken. The origin of the primitive cytoplasm, prebiotic cells and then viable bacterial cell(s) would not have required a macro-scale environment if sufficient water, nutrients and environmental conditions were within the ranges suitable for the origin of life at microscopic dimensions (e.g., microns or less) or multiple microscopic locations, possibly even adjacent to each other. One hypothesis is that the origin of life occurred in an aqueous-hydrocarbon mixture [17] with a size domain in the micron order of magnitude, attached or stabilized on, or between mineral surfaces [18]. In a recent chapter Trevors *et al.* [18] also examined the possible environmental locations for the origin of life and discussed the origin of life on mineral surfaces in a hydrogel and the transition to a biofilm. The next section extends the discussion by trying to understand the composition and organization of the first cytoplasm prior to the origin of the first bacterial cell(s) capable of growth and division.

## 3. Hypothesized Composition and Structure of the First Cytoplasm

The composition of the first cytoplasm (precytoplasm) may have been a simple uncontained gel that progressed to a contained gel cytoplasm within a primitive stable, membrane boundary with some organization, to a more structured cytoplasm just prior to the first bacterial cell division. The initial composition of the precytoplasm, and the composition of the cytoplasm at the first cell division may have been very different. The latter cytoplasm would have been more organized, and contain all necessary cellular mechanisms for growth and cell division, possibly as a minimal cell with a minimal or core genome.

The initial cohesive, structured, organizing cytoplasm would only need to be in the order of about 10^−16^ L, eventually surrounded by a primitive membrane in the order of about 10^−9^ m thick (or in width). These magnitudes may have been less in a primitive nanocell. The point being presented is that the amount of cytoplasm required for the origin of life was in the microscopic molecular range. A molecular scale, cohesive, cytoplasm would be sufficient. To extend this argument, the number of microscopic sub-sites contained in 1 mL of precytoplasm would be immense. This may have increased the number of sites in the cohesive gel or per cytoplasm that could potentially become more organized, enclosed in a stable membrane and then make the transition to a single living bacterial cell. Cohesive water and possibly an oil-water interface or emulsion was the original source of precytoplasm that could trap and retain (via diffusion) sufficient concentrations of small molecules such as potassium, sodium, calcium, magnesium, iron, manganese, zinc, molybdenum, copper and phosphate as some examples, required for the first living cells. Amino acids and eventually proteins, DNA, RNA and ribosomes would become part of the organizing cytoplasm enclosed by a simple but stable cytoplasmic membrane. The ion sources would be available from local geochemical sources. This could all occur without any organic, genetic instructions in the form of DNA and/or RNA. The source, organization and composition of precytoplasm were steps in the organization of matter during progression to the origin of bacterial life.

Another challenge is to elucidate the origin of specific and correct organic genetic instructions in the cytoplasm, where the message carried in the instructions was encoded and decoded during transcription and translation, and the proteins produced were essential for cell growth and division. A primitive but organizing cytoplasm is the best location for this to occur, *i.e*., the origin of organic genetic instructions in a stable, contained precytoplasm. What is now sought is a natural mechanism for the origin of the correct organic, genetic instructions that lead to the regulated and controlled assembly of bacterial cells capable of growth and division, and capable of specific gene expression under changing environmental conditions, with subsequent Darwinian evolution.

The microscopic precytoplasm gel environment does not cause diffusion limitations/problems for gases and molecules, but provides a plausible hypothesis to one part of the origin of bacterial life puzzle-a stable organizing microscopic environment in a sufficient size domain for a minimal cell and eventually a minimal core genome. In addition, there would have been some protection from dehydration, buffering against extreme pH, temperature and osmotic changes, permitting some solar radiation to enter but also offering some protection, buffering against decomposition reactions and favoring polymerization reactions needed in cell organization processes. The precytoplasm structure also confines the molecules to a microscopic location and maybe even countless microscopic precytoplasmic locations. A gel type organizing precytoplasm may have been crucial to the organization of prebiotic cells as the environment would be conducive to the stabilities of nucleic acids, proteins, lipids and the ability of the water in the gel to form 3-D molecular networks that are hydrogen bonded. Osmotic regulation and the capacity to overcome fluctuations in the external higher entropy environment would have been crucial to both the origin of primitive cytoplasm and subsequently cells capable of growth and division. Any event that removed water from the precytoplasm could cause dehydration and impede the organization of the first precytoplasm and prebiotic cell(s).

Inward and outward diffusion into the precytoplasm would also be affected once the first primitive membrane was present. This type of prebiotic cytoplasm or gel environment offers advantages without requiring an energy source, active transport processes, a cytoplasmic membrane, or the initial presence of genetic instructions. Local, microscopic entropy is reduced as the organizing precytoplasm becomes less random (lower entropy) than the more random surrounding environment. Once the microscopic entropy starts to decrease and continues to decrease via prebiotic organization, the possibility of a living cell increases as the local microscopic conditions start to resemble or take on some characteristics of an actual living cell, and not randomness.

The transition (not evolution yet) to a more organized, lower entropy, prebiotic cell and the organizing progression to a cell capable of growth and division, would require core biochemical metabolism with reactants/products, ribosomes, and growth; all dependent on the correct, organic, genetic instructions. mRNA would also be able to diffuse within the microscopic cytoplasm distances to easily contact ribosomes. The pH of the cytoplasm would be more stable, less prone to rapid shifts, and in the range required for the cell biochemistry. This can all be better achieved within an organizing, microscopic cytoplasm with lower entropy than the surrounding environment. The mechanism(s) for organization in the absence of genetic instructions are still unknown; however the precytoplasm gel solves challenges for the origin of prebiotic cells and then life.

It has also been hypothesized that a hydrophobic medium (HM) of hydrocarbons was a possible environment for the origin of bacterial life on Earth [6,17]. The origin of the hydrocarbons in the HM was polymerization of methane in the early atmosphere catalyzed by ultraviolet irradiation [17] which then descended as precipitation to the Earth and produced a hydrophobic hydrocarbon medium. The HM would be conducive to polymerization reactions as opposed to hydrolysis reactions in water that would cause decomposition, not polymerization, and molecular organization.

## 4. Summary and Outlook

Evolutionary theory and current research does not explain the enigma of the organization and emergence of life on the Earth, or elsewhere. It does, however, explain with abundant supporting evidence, the evolution of life after the first living bacterial cells capable of growth and division and mutations and gene transfer (transduction, conjugation, transformation) are present, with natural selection exerting changing selective pressures. It has even been hypothesized that programmed cell death is a necessary prerequisite for multicellularity [19]. Controlled death has advantages over accidental death as it provides a controlled mechanism to remove aging individuals, and resources become available for younger generations. However, cell death in bacteria occurs when environmental conditions are not tolerated (outside the tolerated ranges) by the bacterial cells.

The open question of how physical and chemical activities transformed inanimate molecules of the Earth’s mass (or possibly mass anywhere else in the universe) into enclosed, microscopic cytoplasm and then bacterial cells capable of growth and division is still an immense research challenge. The research community is hampered in a profound way by the inability to conduct some experiments and make observations. This is hopefully changing with the use of synthetic biology approaches [20] to design and hopefully generate prokaryotic cells capable of growth and division.

It is recognized that the hypothesized origin and composition of precytoplasm and prebiotic cells prior to living bacterial cells, requires supporting/non-supporting observations, and experimentation, if and when possible. One research challenge, among many, is not knowing the composition of the primitive cytoplasm, and if it was strictly aqueous, more gel-like or a water-oil emulsion stabilized in some manner on and between mineral surfaces.

Lipids (glycerol bonded to fatty acids and other groups such as phosphate; fatty acids have both hydrophobic-water repelling and hydrophilic regions that are water soluble) may have preceded polypeptides and nucleic acids, as they are able to self-organize into cell-like structures with an inside and outside. If prebiotic life and then living cells had one early requirement, it was a compartment with internal precytoplasm spatial organization and then regulation [21]. This suggests that membranes may have been one of the first components of the self-organizing cellular structure. Biological membranes have numerous functions including creating an inside and outside (cellular compartment), energy generation and storage in the mechanisms of oxidative phosphorylation and photosynthesis) ion and nutrient transport and enzyme-catalyzed reactions [22]. Moreover, simple primitive membranes could have formed without any enzyme catalysis and remained stable enough for subsequent self-organization followed by more biochemical optimization. Lipid and lipid-like molecules are capable of undergoing spontaneous formation of droplets, micelles, bilayers and vesicles in an aqueous medium or at the interface between an aqueous medium and the atmosphere [22]. The vesicle is composed of millions of molecules held together by non-covalent interactions [22]. They also have a significant characteristic central to the self-organization of life; the vesicles have long life times which would be necessary for the self-organization of primitive cell(s).

Interstellar clouds can be viewed as factories of complex molecular synthesis [23] and molecules used in living biochemistry are present in planetary atmospheres, surfaces, meteorites, comets and asteroids that were delivered to the early Earth via impact events [23]. These can be thought of as part the molecular toolbox required for the organization and composition of the first life on the Earth. Especially important would be precytoplasm and membranes capable of organizing into a stable, boundary structure (primitive membrane) such as those reported on by Namani and Deamer [24] made from a 1:1 mole ratio of decyclamine and decanoic acid. Such a stable membrane with increased stability would have an immense advantage over a purely fatty acid primitive membrane in the origin of life. A stable boundary layer such as a membrane enclosing precytoplasm was the type of structure required for the organization of a cell which can be described as a gel [25] where macromolecules such as proteins, and ions have a significant role in structuring/organizing intracellular water which assists in the organization of soft matter-the cell [25].

In addition, microscopic gels may have acted in an interactive manner by combining or exchanging their contents by diffusion and mechanical mixing, thus providing access to the molecules and suitable concentrations (without being depleted) required for the emergence of life on Earth. The progression hypothesized is a prebiotic gel or precytoplasm→more organized precytoplasm→cytoplasm in first living cell(s)→cell cycle (period from one cell division to the next cell division) (bacterial cells capable of duplication of cytoplasm and genetic material, and the subsequent distribution of both into two offspring cells) with local, microscopic entropy decreasing in the cells compared to the higher entropy external environment. The exact time scales required for the origin of bacterial life and the first cell cycles are still not known.

Research on the first prebiotic organization in the absence of genetic instructions and cell structures is a challenge because experiments may be difficult to design and implement. Moreover, the experiments may not necessarily help elucidate the transition from the non-living to the first living cell. There may have been numerous ways for life to originate and the laboratory synthetic biology approach could be different from what actually occurred in the past on the Earth and/or elsewhere.

## Figures and Tables

**Table 1.** Some plausible features of the composition and organization of the first prebiotic cytoplasm (or precytoplasm) and eventually, cytoplasm in the first living bacterial cells capable of growth and division.

■ The prebiotic cytoplasm and cytoplasm in present day bacterial cells is a 3-D biosystem, with some activities now localized to cellular zones [21].
■ A cytoplasm volume in the order of 1 μm^3^ or less is more than sufficient for all nucleic acids, lipids, proteins, other macromolecules, molecules, ions and ribosomes. A spherical bacterial cell structure of about 250–300 nm in diameter is sufficient for a viable cell capable of division.
■ Diffusion (and osmosis of water) over short distances (microns) was possible and sufficient.
■ Clustering of prebiotic molecules (molecular crowding, molecular organization) was plausible; the beginning of early spatial organization in the absence of organic, genetic instructions.
■ The initial bacterial cell shape was likely spherical, as cytoskeleton proteins were not present and necessary.
■ The prebiotic bacterial cytoplasm was possibly a gel structure (structured water).
■ Prebiotic cytoplasm provided a stable micro-environment for the organization of the prebiotic and then living cell(s).
■ No internal membranes were present or necessary.
■ Genetic material could be compacted within the microscopic cytoplasm.
■ Coiling of the DNA was also possible in the cytoplasm of prebiotic cells and the cytoplasm in the first living cell(s).
■ The amount of organic, genetic, instructions required for a microscopic volume of cytoplasm and the first viable bacterial cell(s) was a small core or minimal genome of several hundred genes.
■ Bacterial cytoplasm can contain up to about 76,000 ribosomes/cell [14]; a sufficient number and close enough to the transcribed mRNA for rapid translation to occur. The mRNA contacts ribosomes by diffusion in the cytoplasm. No cytoplasmic streaming is required. Spatial crowing is beneficial to this activity.
■ Ribosomes because of their large sizes likely do not diffuse or move much in the bacterial cytoplasm. However, lower molecular weight transcripts can diffuse short distances to the ribosomes in the absence of cytoplasmic streaming.
■ Precytoplasm in prebiotic cell structures and cytoplasm in living cells(s) were part of an organizing, open, thermodynamic biosystem with lower entropy (less randomness) than the surrounding environmental entropy (more random).
■ Internal osmolality of cytoplasm was becoming more stabilized in the precytoplasm.
■ Ions (e.g., potassium, calcium, manganese, magnesium) can enter the prebiotic cytoplasm or gel by diffusion and be trapped, and made available for cellular organization.
■ Polarization processes (eventually needed for regulated mid-cell bacterial division) are possible in prebiotic cytoplasm especially once the correct genetic instructions are present.
■ Prebiotic cytoplasm could remain stable over a temperature and pH range, and provided some protection from UV irradiation.
■ Diffusion of gases into and exiting prebiotic cytoplasm was possible.
■ Interfaces between aqueous and oily hydrocarbon microscopic environments was possible and even emulsions when turbulence or mechanical mixing was present.
■ Enzymatic and non-enzymatic biochemical reactions would be possible in prebiotic cytoplasm. Enzymes when present provide speed and direction to the organizing biochemistry. Both speed and direction are needed for regulated gene expression and to allow rapid generation times in bacterial cells.
■ Dessication is decreased in a gel-like, prebiotic cytoplasm.
■ Cytoplasm is synthesized and then partitioned during bacterial cell division. Prebiotic cytoplasm could have been physically partitioned.
■ Positive macromolecular charges and small cations only partially neutralize lipid and macromolecular negative charges. The result is a negative stabilization of cytoplasmic macromolecules [5].
■ Energy transduction occurs across a membrane-enclosed cytoplasm.
■ Origin of the bacterial wall and cytoplasmic membrane (CM) during the emergence of the first bacterium capable of growth and division is still an enigma.
■ The spatial organization of bacterial cells requires a better understanding of the mechanisms that localize proteins to specific sites at the necessary concentrations and at specific times [1].
■ Whole genome expression analysis is now possible in bacterial cells exposed to numerous diverse environmental conditions.
■ Can a complete and functional synthetic bacterial cytoplasm and then synthetic bacterial cells be produced?
■ Do so many different bacterial cell parts work as an integrated biosystem over different time scales? The answer is organization, the correct gene instructions expressed at the correct times for the correct time durations under conditions that are within the ranges tolerated by bacteria. The details still need to be better understood.
